# Skeletal versus conventional intraoral anchorage for the treatment of class II malocclusion: dentoalveolar and skeletal effects

**DOI:** 10.1186/s40510-014-0043-z

**Published:** 2014-07-30

**Authors:** Lisa Mariani, Giuliano Maino, Alberto Caprioglio

**Affiliations:** Private Practice of Orthodontics, Via S.Pellico, 79/b, Arcitate, 21051 Varese Italy; Insubria University, Via Valleggio, 11, Como, 22100 Italy; Ferrara University, Via Savonarola, 9, Ferrara, 44121 Italy; Private Practice of Orthodontics, Viale Milano 53, Vicenza, 36100 Italy; Department of Orthodontics, School of Dentistry, University of Insubria, Varese, 22100 Italy; Private Practice in Orthodontics, Via San Zeno 1, Pavia, 27100 Italy

**Keywords:** Molar distalization, Pendulum, Tad's, Class II malocclusion

## Abstract

**Background:**

The purpose of this retrospective study is to investigate the dentoalveolar and skeletal effects of two distalizing protocols featuring different anchorage systems used in patients with class II malocclusion: the MGBM system (skeletal anchorage) and Pendulum (intraoral anchorage).

**Methods:**

The sample comprised 57 patients who were assigned to one of the two treatments: the MGBM group (30 patients, mean age 13.3 ± 2.3 years) or the Pendulum group (27 patients, mean age 12.8 ± 1.7 years). Three serial cephalograms were obtained at baseline (T0), after molar distalization (T1), and after fixed appliance treatment (T2). Esthetic, skeletal, and dental parameters were considered. Pancherz's superimposition method was used to assess sagittal dental changes. The initial and final measurements and treatment changes were compared by means of a paired *t* test or a paired Wilcoxon test. Statistical significance was tested at *p* < 0.05, *p* < 0.01, and *p* < 0.001.

**Results:**

In the MGBM group, the upper molar distalization was achieved in 7 months and showed a mean value of 4.9 mm (ms-PLO); the amount of molar relationship correction was 5.9 mm. In the Pendulum group, the upper molar distalization was obtained in 9 months and showed a mean value of 2.5 mm (ms-PLO), while the molar relationship correction amounted to 4.9 mm. Anterior anchorage loss occurred in both groups, although in the MGBM group, there was less mesial movement of the premolars.

**Conclusions:**

The MGBM system and the Pendulum appliance are both effective in the correction of class II malocclusions. The MGBM system was found to be more efficient than the Pendulum appliance, producing greater molar distalization in a shorter treatment time.

## Background

Upper molar distalization is a common orthodontic procedure often required for the treatment of class II malocclusions. Several distalization devices have been widely used as the main alternatives to extraction treatment [1,2]. Even though they are effective means of achieving tooth movement, all these treatments are highly dependent on patient compliance. Beginning in the 1990s, many treatment protocols have been suggested with a view to reduce this dependence on patient compliance [3,4].

All these procedures effectively distalize both upper molars, but may cause anchorage loss as a result of using palatal buttons or premolar anchorage arms. Recently, researchers have tried to overcome this problem by designing new intraoral systems involving skeletal anchorage solutions.

In 2011, Fudalej and Antoszewska [5] conducted a systematic review of 12 relevant articles describing distalizing appliances reinforced with temporary skeletal anchorage devices in order to study their efficacy. They concluded that ‘Molar distalizers reinforced with the temporary skeletal anchorage devices seem to effectively move molars distally without unwanted mesial incisor tipping’.

**Figure 1:**
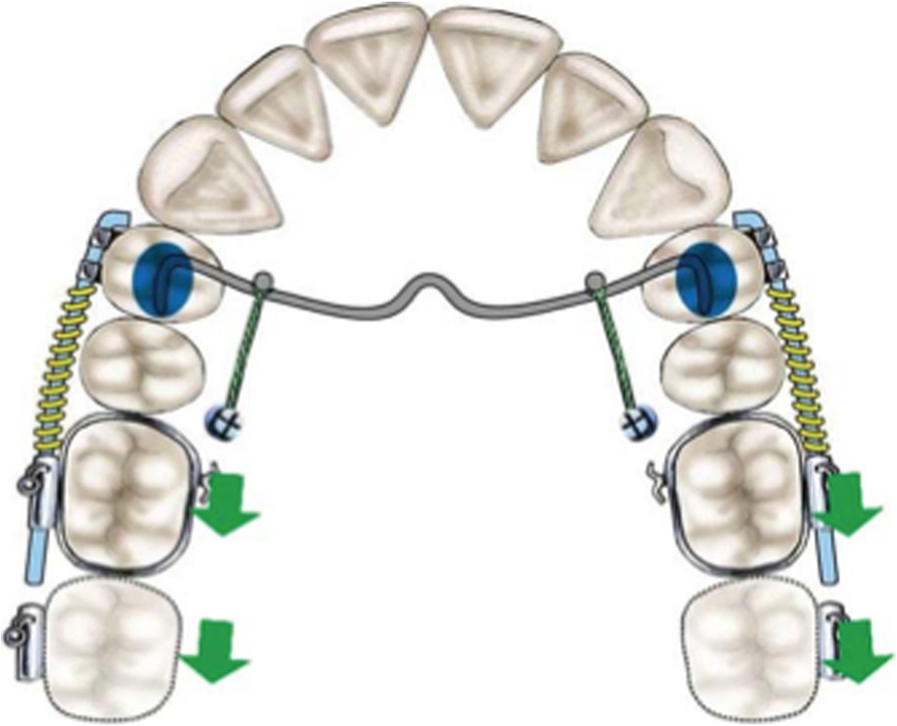
**MGBM system (palatal anchorage).**

**Figure 2:**
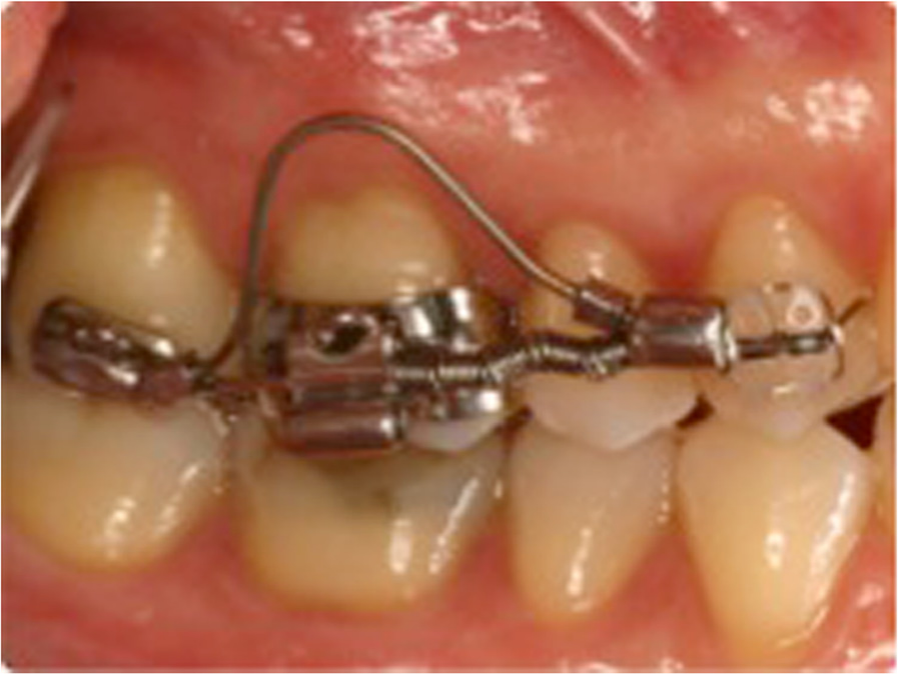
**MGBM system (distalizing mechanics).**

**Figure 3:**
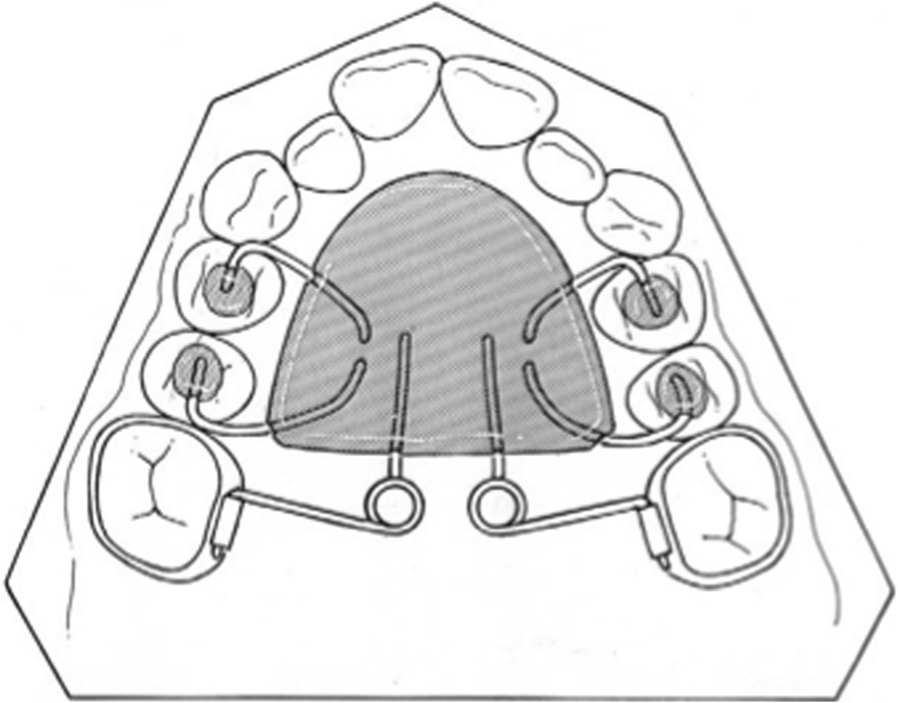
**Pendulum.**

**Figure 4:**
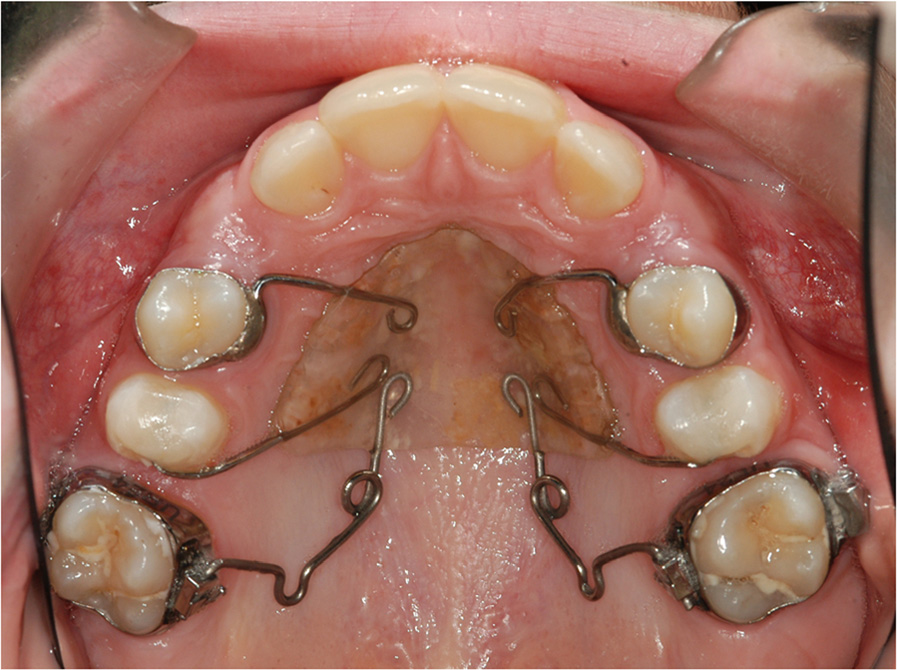
**Pendulum (distalizing mechanics).**

The purpose of this retrospective study was to investigate the dentoalveolar and skeletal effects of two distalizing protocols featuring different anchorage systems used in patients with class II malocclusion: the MGBM system [6] (Figures 1 and 2) and the Pendulum appliance [7] (Figures 3 and 4).

## Methods

Three serial cephalograms of 57 patients were obtained at baseline (T0), after molar distalization (T1), and after fixed appliance treatment (T2). Thirty of these patients (age 13.3 ± 2.3 years) were treated with the MGBM system, and the remaining 27 (age 12.8 ± 1.7 years) were treated with Pendulum. The two groups of patients were treated consecutively by two specialists and board-certified experts, respectively, in one of the two techniques. The selection criteria included were the following:

- Skeletal class I or II malocclusion (angle classification) and bilateral full cusp or end-to-end class II molar relationship with moderate space deficiency in the maxillary dental arch, minimal or no crowding in the mandibular arch;
- Permanent dentition;
- Patients at pubertal growth spurt (stage C3 or C4) [8];
- SN/GoGn angle less than 37°;
- T0 to T1 > 12 months;
- Non-extraction treatment;
- Intermaxillary elastics used only after molar distalization (during fixed multibracket therapy);
- Good-quality radiographs with adequate landmark visualization.

The records of 14 patients from the whole sample were excluded due to poor film quality or incomplete records, and 3 patients were excluded because the mandibular plane angle was greater than 37°. An additional seven patients were excluded because the time period between T0 and T1 was greater than 12 months, and finally, three patients were excluded because other molar distalizing mechanics were used. Gender differences were not considered a factor because treatment was performed in non-growing patients.

The first phase of treatment (T0-T1) was designed to achieve an overcorrected class I molar relationship, while the second (T1-T2) consisted of fixed appliance therapy to align and detail the occlusion. Intermaxillary elastics were used during multibrackets therapy in both groups.

The mean time period between the initial T0 radiograph and the post-distalization T1 radiograph was 8 ± 2 months and 9 ± 3 months, respectively, for the MGBM system and Pendulum groups. The mean time period between the initial T0 radiograph and the post-treatment T2 radiograph was 2 years and 1 ± 2 months in the MGBM system group and 2 years and 7 ± 5 months in the Pendulum group.

For a description of the devices used, the readers are referred to the authors' original articles [6,7]. In the MGBM system, the anchorage is provided by a transpalatal bar, which is bonded to the occlusal surfaces of the maxillary first premolars and to which two palatal miniscrew, inserted directly between the first molar and the second premolar bilaterally, are connected (Figure 1). It is easy to find the space to insert two miniscrews in this site because palatally, the distance from the palatal root of the first molar and the root of the second premolar is rather large.

A sectional buccal mechanics consisting of a sectional 0.016 × 0.022-in SS wire extending from the first premolar to the first molar and a compressed 200-g Sentalloy coil (DENTSPLY GAC International, Islandia, NY, USA) is used to distalize the first molar. When the second molars are present, a second buccal distalizing component provided by a shape memory 0.018 × 0.025-in NeoSentalloy wire (DENTSPLY GAC International) is inserted between the second molar and the first premolar (looped vertically for 6 mm in the buccal fold) (Figure 2).

The Pendulum appliance used in this study was similar to the original described by Hilgers [7] with bands on the first molars, occlusal rests on premolars, and initial activation of the TMA springs at about 90° (Figures 3 and 4). At the end of distalization, the Pendulum was replaced with a Nance plate, leaving the second premolars free to drift spontaneously in a distal direction. When the second molars are erupted, the Pendulum springs were inserted in the second molars first.

**Figure 5:**
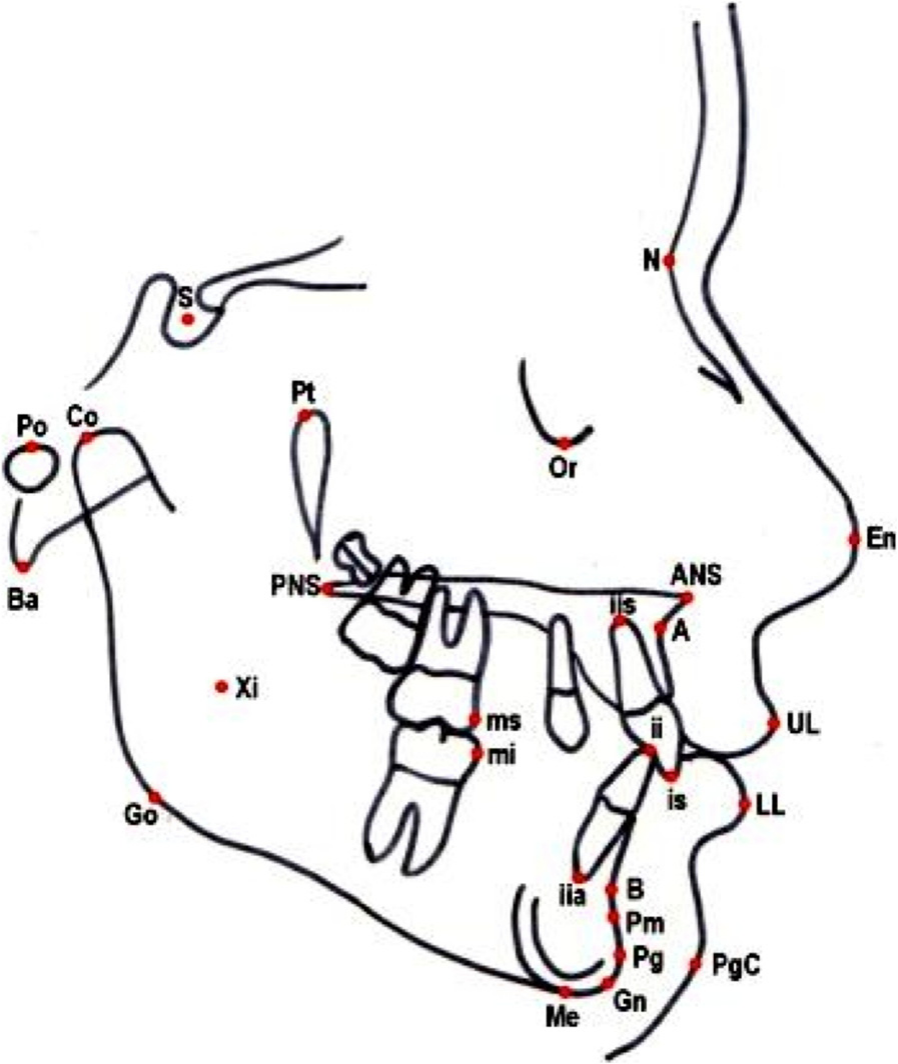
**Cephalometric landmarks.** Co = condilion, Po = porion, S = sella; Ba = basion, Pt = pterygoid point, Or = orbital, N = nasion, En = tip of nose, UL = superior labial point, LL = inferior labial point, Xi = mandibular centroid, ANS = anterior nasal spine, PNS = posterior nasal spine, A = subnasal point, B = sovramental point, Pm = paramedian point, Pg = pogonion, Pgc = soft pogonion, Gn = gnathion, Me = menton, Go = gonion, ii = lower incisor point, is = superior incisor point, iia = apical point of lower incisor, isa = apical point of superior incisors; ms = mesial point of upper first molar, mi = mesial point of lower first molar.

**Figure 6:**
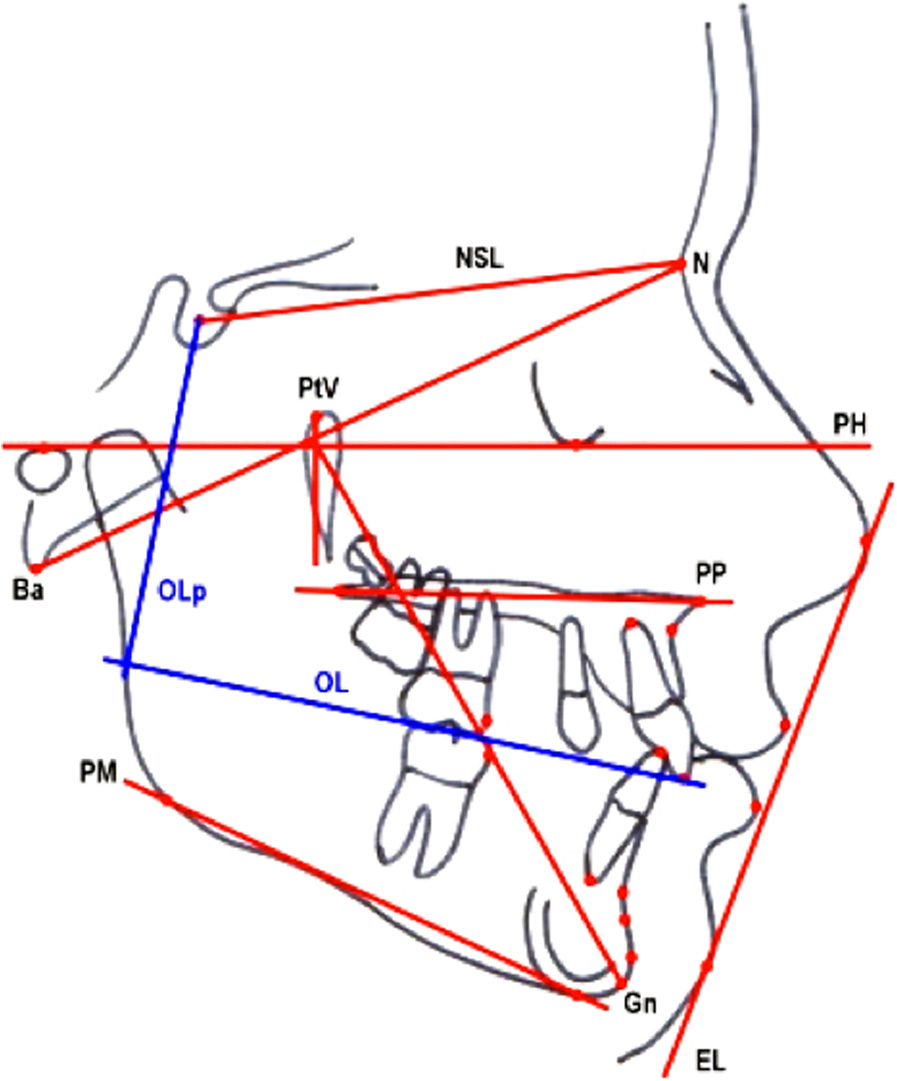
**Cephalometric planes.** NSL = sella-nasion line, PP = palatal plane, PM = mandibular plane, OL = occlusal plane, OLp = perpendicular occlusal plane passing for sella point, Ba-Na = cranio-basal plane, Pt-Gn = facial axis, E-plane = esthetic plane, N-Pg = facial plane.

**Figure 7:**
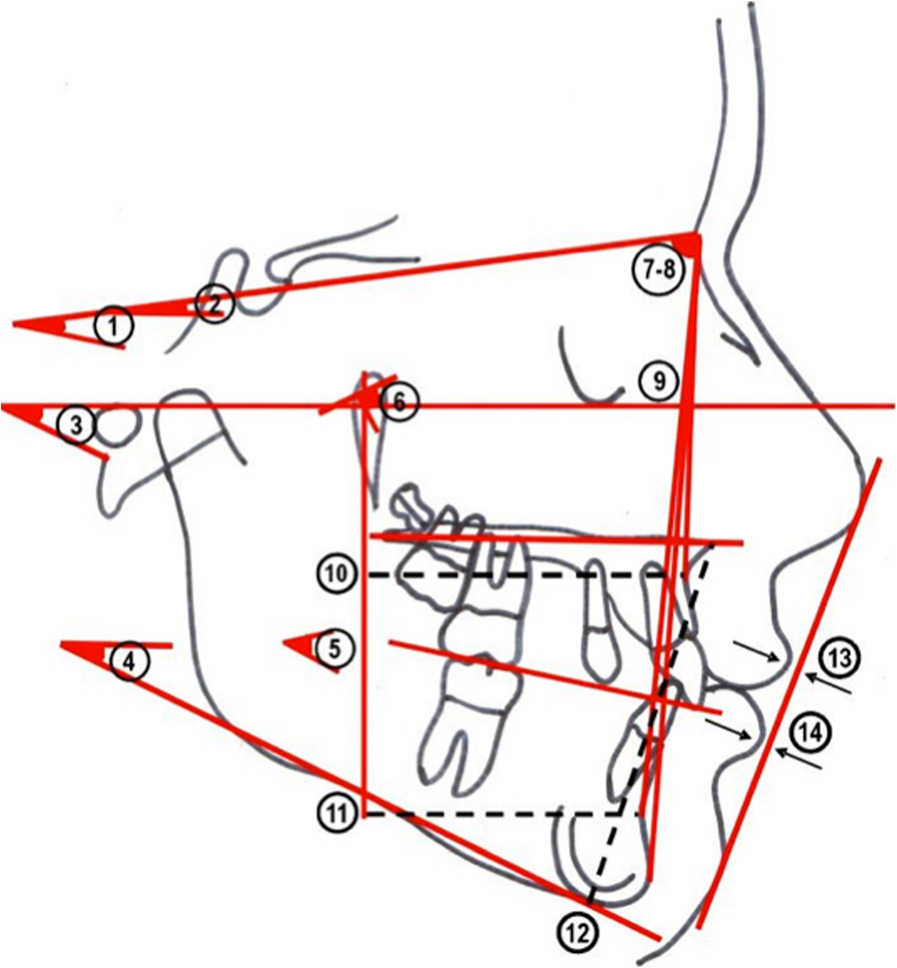
**Skeletal and esthetical measurements.** (1) SN^OL°, (2) SN^PP°, (3) FMA°, (4) GoMe^PP°, (5) ANS-Xi-Pm°, (6) BaN^PtGn°, (7) SNA°, (8) SNB°, (9) FH^NPg°, (10) PTV-A, (11) PTV-B, (12) ANS-Me, (13) UL-E plane, (14) LL-E plane.

**Figure 8:**
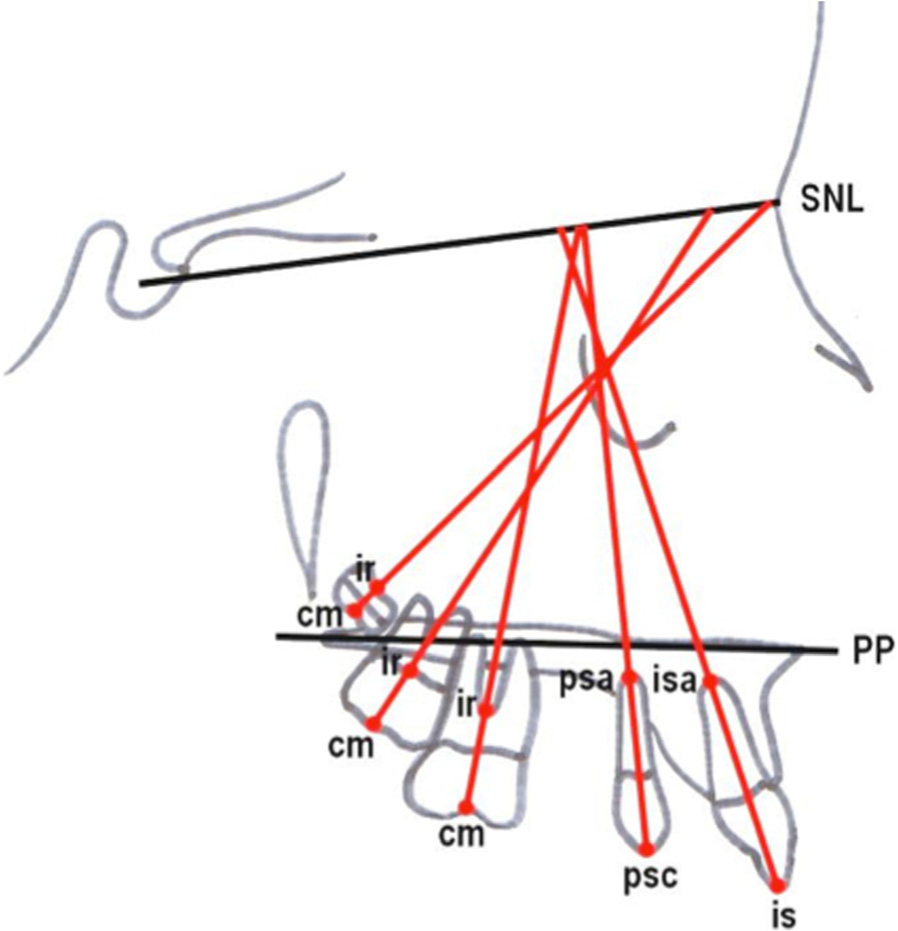
**Angular dental measurements.** (1) SN^1.1 axis (isa-is), (2) PP^1.1 axis (isa-is), (3) SN^1.4 axis (psa-psc), (4) SN^1.6 axis (cm-ir), (5) PP^1.6 axis (cm-ir), SN^1.7 axis (cm-ir), PP^1.7 axis (cm-ir), SN^1.8 axis (cm-ir).

**Figure 9:**
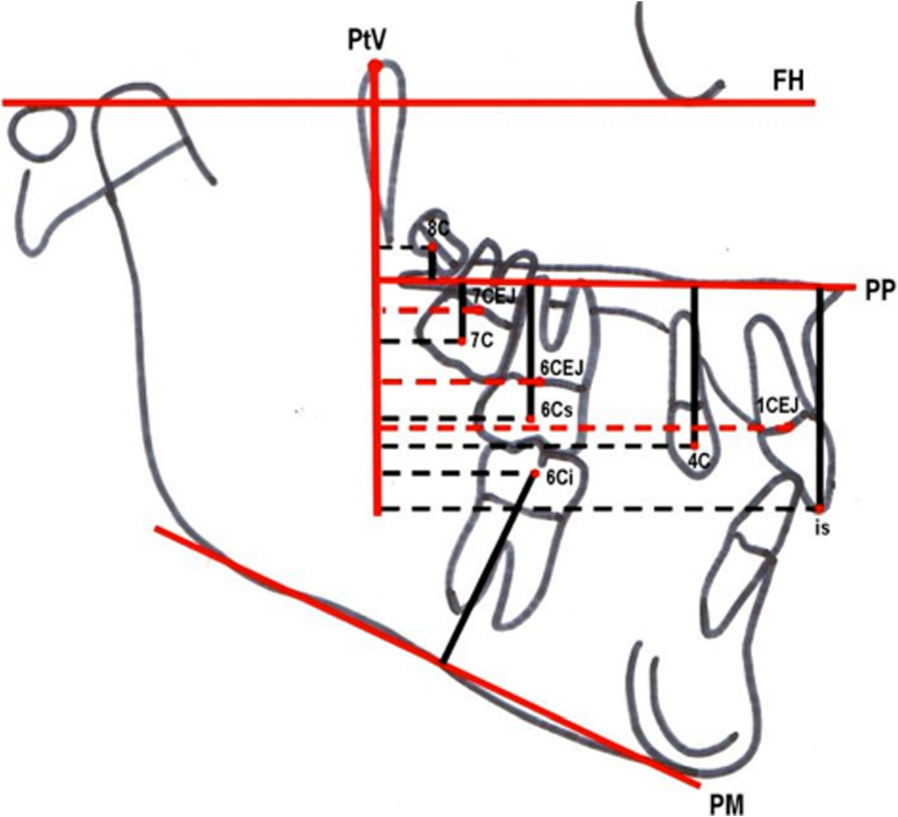
**Linear dental measurements referred to PTV and PP.** (1) PP-is, (2) PP-1.4 centroid, (3) PP-1.6 centroid, (4) PP-1.7 centroid, (5) PP-1.8 centroid, (6) PTV-11 CE, (7) PTV-1.4 centroid, (8) PTV-1.6 CEJ, (9) PTV-1.6 centroid, (10) PTV-1.7 CEJ, (11) PTV-1.7 centroid, (12) PTV-1.8 centroid, (13) PTV-4.6 CEJ.

The cephalometric measurements used were based on those defined and described by Ghosh and Nanda (Figures 5 and 6) [9]. We considered esthetic and skeletal (Figure 7) and angular dental (Figure 8) and linear dental measurements (Figure 9).

**Figure 10:**
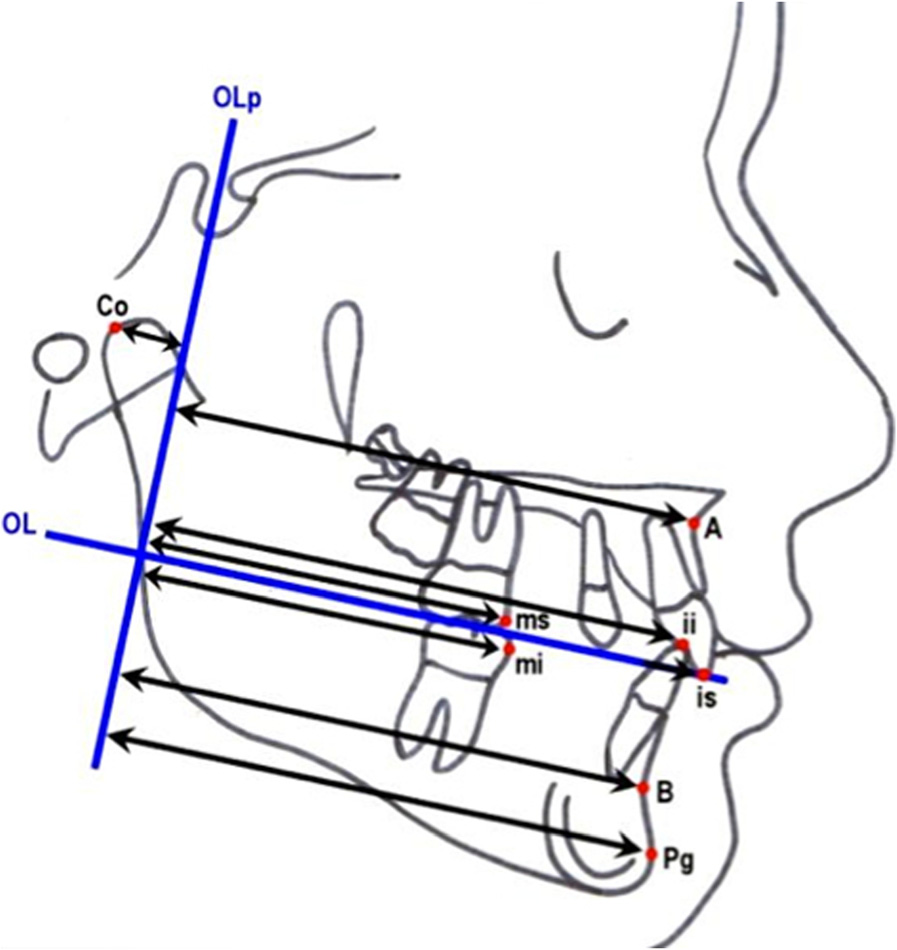
**Cephalometric measurements referred to OLp.** (1) A-OLp, (2) PTV-B, (3) B-OLp, (4) Pg-OLp, (5) Co-OLp, (6) Pg-OLp + Co-OLp.

Pancherz's [10] superimposition method was used to assess sagittal dental changes and to avoid errors due to possible inclination of the occlusal plane after molar distalization (Figure 10). Cephalometric measurements were made to the nearest 0.5 mm or 0.5°. Images of bilateral structures were bisected. Lateral cephalograms for each patient at T0, T1, and T2 in both treatment groups were standardized as to magnification factor (8% enlargement) and digitized.

The main outcome measures to be assessed on the cephalograms were the following:

- Distal movement and distal tipping of maxillary first molars.
- Anchorage loss (anterior movement and proclination of maxillary first premolars and maxillary central incisors).

### Statistical analysis

Statistical analysis was performed using a statistical software package MedCalc version 12.2.1 (Mariakerke, Gent, Belgium). In order to compare the pre-treatment cephalometric data recorded by the two different specialists, a non-parametric Mann-Whitney independent samples test was performed. The small size of the samples precluded the use of a parametric *t* test. No significant differences were found between the two groups. In order to verify the normal distribution of the sample data, a D'Agostino-Pearson test was performed for each cephalometric variable; if normal distribution was detected, a paired *t* test was used to identify significant between-group differences for each cephalometric variable; if normal distribution was rejected, a Wilcoxon test for paired data was used. Statistical significance was tested at *p* < 0.05, *p* < 0.01, and *p* < 0.001.

Lateral cephalographs were traced and measured twice at a 2-week interval by two researchers. If values deviated, the means of both measurements were fed into the statistical analysis. The mean cephalogram measurement error [11] was found to range from 0.09 to 0.3 mm for linear measurements, and from 0.3° to 0.7° for angular measurements, while the reliability coefficient ranged from 0.93 to 0.98 and from 0.92 to 0.98, respectively.

## Results

Table 1 shows the results of the descriptive statistics of the cephalometric values. Multivariate analysis showed no significant differences between the two samples at T0. Descriptive and inferential statistics of changes occurring during the different treatment phases (T0-T1, T1-T2, and T0-T2) are summarized in Tables 2, 3 and 4, respectively.

**Table 1:** **Initial cephalometric values**

	**MGBM **		**Pendulum**	
	**Mean**	**SD**	**Mean**	**SD**
Aesthetical values (mm)				
UL-E plane	−1.8	1.5	−4.5	6.7
LL-E plane	−0.2	2.0	−2.0	2.3
Vertical skeletal values (°)				
SN^PP	8.7	5.7	8.3	4.4
SN^OL	19.3	1.9	19.8	4.3
FMA	21.7	2.1	19.9	3.8
Go-Me^PP	23.7	2.5	22.5	5.2
ANS-Xi-Pm	43.3	3.1	40.2	3.4
Ba-Na^Pt-Gn	87.4	2.9	89.5	0.2
Vertical skeletal values (mm)				
ANS-Me	68.3	1.5	65.3	5.4
Sagittal skeletal values (°)				
SNA	81.0	3.1	81.1	2.9
SNB	76.4	2.3	77.4	2.5
ANB	4.3	2.4	3.6	1.8
FH^NPg	87.9	2.5	89.4	2.4
Sagittal skeletal values (mm)				
PTV-A	53.4	3.1	55.6	2.7
A-OLp	81.2	3.5	81.0	3.5
PTV-B	60.2	3.3	50.6	2.7
B-OLp	77.9	2.6	81.6	3.8
Pg-OLp	84.9	2.7	85.6	3.3
Co-OLp	9.6	3.0	10.0	3.4
Pg-OLp + Co-OLp	94.4	3.3	92.0	2.6
Angular dental values (°)				
SN^1.1 axis (central superior incisor)	103.8	6.9	103.2	8.3
PP^1.1 axis (central superior incisor)	108.7	4.1	107.7	5.7
SN^1.4 axis (first superior premolar)	84.6	6.3	84.0	4.5
SN^1.6 axis (first superior molar)	67.9	6.3	66.9	5.1
PP^1.6 axis (first superior molar)	75.6	4.8	77.6	5.7
SN^1.7 axis (second superior molar)	56.5	4.7	60.8	7.1
PP^1.7 axis (second superior molar)	64.1	6.0	65.2	6.1
SN^1.8 axis (third superior molar)	37.1	9.1	38.0	8.7
Sagittal linear dental values (mm)				
OLp-is	89.6	6.8	89.1	4.0
PTV-11 CEJ	55.9	6.5	56.2	3.6
OLp-ii	84.3	1.5	83.4	3.9
Difference OLp-is - OLp-Ii (overjet)	5.7	5.2	5.7	2.4
PTV-1.4 centroid	39.7	5.8	40.2	3.3
OLp-ms	58.0	2.0	57.3	3.2
PTV-1.6 CEJ	22.5	6.1	22.9	2.9
PTV-1.6 centroid	22.1	4.1	21.4	5.6
PTV-1.7 CEJ	13.3	4.4	13.7	2.7
PTV-1.7 centroid	12.4	4.3	13.1	3.0
PTV-1.8 centroid	8.4	4.4	8.8	2.5
OLp-mi	57.5	4.3	57.2	3.8
PTV-4.6 CEJ	20.4	4.5	21.9	3.8
OLp-ms - OLp-mi	0.2	4.4	0.8	2.0
Vertical linear dental values (mm)				
PP-is	30.4	2.0	30.0	2.8
PP-1.4 centroid	21.8	2.3	21.0	2.8
PP-1.6 centroid	19.0	2.0	17.5	2.8
PP-1.7 centroid	11.5	4.7	0.5	4.6
PP-1.8 centroid	−3.4	2.1	−0.7	3.3
PM-4.6 centroid	29.6	6.5	27.1	2.4

**Table 2:** **Means and standard deviations of cephalometric changes after distalization (T0-T1)**

	**MGBM**		**Pendulum**		***p*** **Value**
	**Mean**	**SD**	**Mean**	**SD**	
Esthetical variation (mm)					
UL-E plane	0.7	0.9	0.5	1.4	0.347
LL-E plane	0.5	1.3	0.6	1.5	0.479
Vertical skeletal changes (degrees)					
SN^PP	0.5	1.1	0.0	2.3	0.275
SN^OL	1.6	1.5	−0.5	2.3	0.000***
FMA	1.2	0.9	0.5	1.5	0.009**
Go-Me^PP	0.5	1.6	0.8	2.1	0.321
ANS-Xi-Pm	1.0	1.4	0.5	1.9	0.524
Ba-Na^Pt-Gn	−0.8	1.0	0.3	2.0	0.006**
Vertical skeletal changes (mm)					
ANS-Me	2.0	1.5	2.0	1.5	0.241
Sagittal skeletal changes (degrees)					
SNA	0.7	0.9	0.0	1.7	0.233
SNB	−0.7	0.9	0.4	2.1	0.009**
ANB	0.5	1.1	−0.5	1.2	0.008**
FH^NPg	−0.2	1.6	0.5	1.9	0.177
Sagittal skeletal changes (mm)					
PTV-A	1.0	1.5	0.1	2.1	0.003**
A-OLp	0.8	1.4	0.7	2.1	0.524
PTV-B	−0.1	2.0	0.8	2.7	0.294
B-OLp	0.2	2.0	1.6	2.1	0.004**
Pg-OLp	0.4	2.2	2.1	2.3	0.005**
Co-OLp	0.5	1.4	0.2	1.9	0.375
Pg-OLp + Co-OLp	0.9	2.2	2.5	2.9	0.008**
Angular dental changes (degrees)					
SN^1.1 axis	1.4	2.5	4.7	3.9	0.000***
PP^1.1 axis	1.2	3.0	4.9	4.5	0.000***
SN^1.4 axis	2.5	4.3	1.9	6.6	0.479
SN^1.6 axis	−10.5	6.2	−10.3	8.4	0.323
PP^1.6 axis	−9.0	7.7	−9.8	9.7	0.347
SN^1.7 axis	−10.1	9.7	−9.0	11.1	0.294
PP^1.7 axis	10.2	7.8	10.4	10.1	0.247
SN^1.8 axis	−8.2	9.6	−9.6	10.9	0.321
Sagittal dental changes (mm)					
OLp-is	1.6	2.0	2.9	2.0	0.043*
PTV-11 CEJ	1.0	1.8	1.5	2.8	0.523
OLp-ii	0.2	3.1	1.8	2.2	0.421
Difference OLp-is - OLp-Ii	1.1	2.4	1.0	2.0	0.213
PTV-1.4 centroid	1.8	2.0	2.7	3.3	0.242
OLp-ms	−4.9	3.1	−2.5	2.1	0.000***
PTV-1.6 CEJ	−4.1	2.8	−2.1	1.8	0.000***
PTV-1.6 centroid	−4.7	2.9	−2.7	3.7	0.000***
PTV-1.7 CEJ	−4.0	2.4	−2.9	2.9	0.248
PTV-1.7 centroid	−4.7	2.2	−3.7	2.8	0.367
PTV-1.8 centroid	−1.4	1.6	−0.2	2.0	0.002**
OLp-mi	1.0	2.0	2.3	3.1	0.002**
PTV-4.6 CEJ	0.8	1.9	1.3	2.6	0.478
OLp-ms - OLp-mi	−5.9	3.4	−4.9	2.5	0.004**
Vertical dental variations (mm)					
PP-is	0.5	1.1	0.5	1.4	0.194
PP-1.4 centroid	1.1	1.9	1.4	1.7	0.172
PP-1.6 centroid	1.3	0.9	0.1	1.6	0.359
PP-1.7 centroid	−0.9	2.1	0.1	2.4	0.043*
PP-1.8 centroid	−1.1	3.0	−2.1	5.9	0.466
PM-4.6 centroid	1.2	0.9	0.9	1.0	0.132

* Implies significance at p< 0.05, ** Implies significance at p< 0.01, *** Implies significance at p< 0.01.

**Table 3:** **Means and standard deviations of cephalometric changes after fixed appliance (T1-T2)**

	**MGBM**		**Pendulum**		***p*** **Value**
	**Mean**	**SD**	**Mean**	**SD**	
Esthetical variations (mm)					
UL-E plane	−2.9	1.1	−2.8	1.8	0.532
LL-E plane	−2.4	2.1	−2.0	1.6	0.736
Vertical skeletal variations (°)					
SN^PP	0.9	1.2	0.5	2.2	0.263
SN^OL	−1.2	2.7	−0.1	2.8	0.000***
FMA	−2.2	1.9	−0.3	1.8	0.003**
Go-Me^PP	0.0	2.1	−0.5	2.3	0.621
ANS-Xi-Pm	−1.8	1.7	0.0	1.3	0.534
Ba-Na^Pt-Gn	2.4	2.1	−0.1	2.5	0.005**
Vertical skeletal variations (mm)					
ANS-Me	0.5	3.0	1.7	2.8	0.572
Sagittal skeletal variations (°)					
SNA	−1.0	1.0	−0.9	2.2	0.826
SNB	0.0	1.1	−0.5	1.7	0.265
ANB	−1.0	1.6	−0.3	1.4	0.165
FH^NPg	3.3	2.2	0.4	2.1	0.006**
Sagittal skeletal variations (mm)					
PTV-A	2.2	2.6	0.4	2.2	0.002**
A-OLp	−0.2	1.5	0.2	2.4	0.243
PTV-B	3.8	3.1	1.0	2.6	0.000***
B-OLp	0.5	2.3	1.0	2.5	0.132
Pg-OLp	1.5	3.4	1.9	3.4	0.534
Co-OLp	0.7	1.9	1.0	1.9	0.652
Pg-OLp + Co-OLp (mandibular length)	1.9	3.2	3.1	3.4	0.735
Dental angular variations (°)					
SN^1.1 axis (central superior incisor)	−2.6	5.9	−5.0	7.8	0.002**
PP^1.1 axis(central superior incisor)	−2.9	5.8	−3.9	9.7	0.023*
SN^1.4 axis (first superior premolar)	0.6	5.0	−6.3	5.8	0.003**
SN^1.6 axis (first superior molar)	12.2	8.3	9.1	9.1	0.365
PP^1.6 axis (first superior molar)	12.1	7.9	12.1	7.6	0.765
SN^1.7 axis (second superior molar)	14.9	9.6	11.6	13.3	0.635
PP^1.7 axis (second superior molar)	15.6	9.3	16.8	9.8	0.271
SN^1.8 axis (third superior molar)	5.4	9.6	8.2	17.9	0.127
Dental sagittal variations (mm)					
OLp-is	−1.6	2.1	−1.4	3.6	0.142
PTV-11 CEJ	3.7	3.4	0.2	3.1	0.004**
OLp-ii	−1.6	2.1	2.4	3.4	0.002**
Difference OLp-is - OLp-Ii (overjet)	−3.1	2.1	−3.8	2.7	0.345
PTV-1.4 centroid	0.1	2.9	−1.4	3.2	0.763
OLp-ms	4.0	1.2	4.0	2.8	0.654
PTV-1.6 CEJ	5.1	2.1	3.4	3.8	0.354
PTV-1.6 centroid	5.0	4.9	4.5	4.1	0.874
PTV-1.7 CEJ	4.3	2.3	2.5	3.8	0.263
PTV-1.7 centroid	5.7	3.2	4.5	4.3	0.162
PTV-1.8 centroid	3.0	2.3	0.4	1.8	0.635
OLp-mi	1.2	1.9	3.2	3.9	0.324
PTV-4.6 CEJ	4.3	3.0	3.9	2.5	0.532
OLp-ms - OLp-mi (molar relationship)	2.2	4.3	1.4	1.9	0.625
Vertical dental variations (mm)					
PP-is	−1.1	1.3	0.8	2.3	0.004**
PP-1.4 centroid	−0.2	2.0	−0.3	2.4	0.253
PP-1.6 centroid	0.5	2.2	2.2	2.7	0.02*
PP-1.7 centroid	4.6	4.7	4.0	3.4	0.127
PP-1.8 centroid	4.6	0.6	5.7	6.4	0.138
PM-4.6 centroid	2.5	2.9	2.0	1.8	0.142

* Implies significance at p< 0.05, ** Implies significance at p< 0.01, *** Implies significance at p< 0.01.

**Table 4:** **Means and standard deviations of cephalometric changes after orthodontic treatment (T0-T2)**

	**MGBM**		**Pendulum**		***p*** **Value**
	**Mean**	**SD**	**Mean**	**SD**	
Esthetical changes (mm)					
UL-E plane	−2.5	0.35	−2.09	1.7	0.339
LL-E plane	−2.4	1.12	−1.37	1.6	0.142
Vertical skeletal changes (°)					
SN^PP	1.7	1.22	0.66	1.8	0.190
SN^OL	−0.2	3.06	−0.06	2.8	0.526
FMA	−0.5	1.58	0.61	2.0	0.249
Go-Me^PP	0.5	2.51	0.41	2.5	0.479
ANS-Xi-Pm	−0.1	1.78	1.02	1.7	0.178
Ba-Na^Pt-Gn	1.5	1.23	0.09	3.0	0.343
Vertical skeletal changes (mm)					
ANS-Me	3.2	3.21	4.15	2.6	0.198
Sagittal skeletal changes (°)					
SNA	−0.2	1.8	−1.0	2.2	0.176
SNB	−1.2	1.0	−0.4	1.7	0.526
ANB	0.0	1.5	−0.5	1.3	0.248
FH^NPg	1.7	2.1	0.6	1.6	0.354
Sagittal skeletal changes (mm)					
PTV-A	2.5	2.1	0.6	2.5	0.021*
A-OLp	1.2	1.9	1.1	2.3	0.372
PTV-B	3.1	2.3	1.6	2.8	0.191
B-OLp	1.1	2.4	2.4	2.6	0.254
Pg-OLp	2.4	2.8	4.1	3.5	0.543
Co-OLp	0.9	2.1	0.2	1.8	0.469
Pg-OLp + Co-OLp (mandibular length)	2.9	2.8	4.3	3.9	0.357
Dental angular changes (°)					
SN^1.1 axis (central superior incisor)	−3.3	6.7	−0.3	7.3	0.354
PP^1.1 axis(central superior incisor)	−0.7	6.8	2.3	10.3	0.479
SN^1.4 axis (first superior premolar)	1.2	5.0	−2.8	7.9	0.198
SN^1.6 axis (first superior molar)	1.3	4.9	−0.3	6.2	0.276
PP^1.6 axis (first superior molar)	3.2	5.7	0.5	6.8	-
SN^1.7 axis (second superior molar)	0.6	9.0	4.4	10.0	0.456
PP^1.7 axis (second superior molar)	2.5	5.9	8.4	9.5	0.321
SN^1.8 axis (third superior molar)	2.3	10.9	3.5	18.3	0.523
Sagittal dental variations (mm)					
OLp-is	−0.1	2.9	0.9	3.1	0.365
PTV-11 CEJ	4.2	3.3	1.1	3.3	0.041*
OLp-ii	1.8	2.6	2.9	3.7	0.236
Difference OLp-is - OLp-Ii (overjet)	−2.0	1.2	−2.5	2.4	0.527
PTV-1.4 centroid	0.8	2.4	1.4	3.3	0.376
OLp-ms	−1.0	3.0	1.8	2.1	0.008**
PTV-1.6 CEJ	−0.5	2.7	1.4	2.9	0.842
PTV-1.6 centroid	0.5	4.5	2.0	4.3	0.642
PTV-1.7 CEJ	−0.5	1.9	0.3	2.3	0.254
PTV-1.7 centroid	0.4	2.5	1.1	2.7	0.415
PTV-1.8 centroid	1.3	2.6	−0.1	1.7	0.451
OLp-mi	2.7	2.6	4.8	3.2	0.721
PTV-4.6 CEJ	4.7	2.8	4.6	3.2	0.823
OLp-ms - OLp-mi (molar relationship)	−3.9	2.4	−2.9	2.0	0.421
Vertical dental changes (mm)					
PP-is	−0.8	1.9	1.4	1.7	0.007**
PP-1.4 centroid	0.6	2.3	1.3	1.6	0.634
PP-1.6 centroid	−0.4	2.1	2.1	1.8	0.009**
PP-1.7 centroid	3.8	4.7	4.1	3.8	0.376
PP-1.8 centroid	1.9	3.5	3.9	3.1	0.243
PM-4.6 centroid	4.7	2.6	2.9	1.6	0.215

* Implies significance at p< 0.05, ** Implies significance at p< 0.01, *** Implies significance at p< 0.01.

### Pre-treatment to post-distalization

The distalization procedure was effective in both groups, with all the patients achieving a class I molar relationship. In the MGBM group, upper molar distalization was achieved in 8 ± 2.05 months, showing mean values of 4.9 mm (ms-PLO), 4.1 mm (pterygoid vertical (PTV)-6 CEJ), and 4.7 mm (PTV-6C); the correction of the molar relationship was 5.9 mm. In the Pendulum group, upper molar distalization took longer (9 months) and showed significantly lower mean values (*p* = 0.005): 2.5 mm (ms-PLO), 2.1 mm (PTV-CEJ), and 2.7 mm (PTV-6C), while the molar relationship was corrected by 4.9 mm. The distal movement of the second upper molar was found to be similar to that of the first molar: 4.0 mm (PTV-7 CEJ) and 4.7 mm (PTV-7C) in the MGBM group versus 2.9 mm (PTV-7CEJ) and 3.7 mm (PTV-C) in the Pendulum group. However, unlike the first molar, the distal movement of the second molar was not significant in either group (*p* = 0.5). The maxillary molar crowns tipped distally by a mean of 10.5° (SN) and 9° (PP) in the MGBM group and 8.3° (SN) and 9.8° (PP) in the Pendulum group. Moreover, the MGBM group also showed 1.3-mm extrusion of the upper first molars. Anterior anchorage loss occurred in both groups, although the MGBM group recorded less mesial movement of the premolars compared with the Pendulum group.

As seen on the cephalograms, the maxillary first premolars were tipped mesially by 2.5° in the MGBM group and 1.9° in the Pendulum group. However, the differences in distal tipping and in the loss of anchorage in the two groups were not statistically significant. The maxillary incisors proclined by an average of 1.4 ± 2.5° and 4.7° ± 3.9°, and advanced by an average of 1.6 ± 2 and 2.9 ± 2 mm in the MGBM and Pendulum groups, respectively. The mean difference in incisor position was statistically significant between the two groups.

Significant changes in the skeletal vertical dimension were higher in the MGBM group than in the Pendulum group with differences of 1.0°, 0.4°, and 0.2°, respectively, for SN^OLp, FMA, and (BaNa^PtGn).

### Post-distalization to the end of orthodontic treatment

Both groups recorded a reduction in the inclination of the maxillary incisors thanks to the fixed appliance treatment (1.1^SN: −2.6° MGBM, −5.0° Pendulum). No significant differences between the two groups were found in vertical and horizontal skeletal relationships. In this phase, the maxillary first molars showed the same amount of mesial movement both in the MGBM- and in the Pendulum-treated patients. However, the Pendulum group, compared with the MGBM group, showed significantly greater mesial tipping of premolars and less upper incisor intrusion.

### Overall treatment effects

In both groups, dentoalveolar correction of class II malocclusion was obtained. No significant differences were found in vertical and sagittal relationships, with the exception of the A point, which proved to be more forward-projected in the MGBM group (Δ PTV-A, 2.5 mm MGBM vs. 0.6 mm Pendulum; *p* = 0.04). Similarly, the upper incisors were less retruded in the MGBM group (Δ PTV −1.1 CEJ, 4.2 mm in MGBM vs. 1.1 mm in Pendulum; *p* = 0.03). At the end of treatment, the MGBM group showed significantly greater molar distalization than the Pendulum group (Δ PLO-ms, −1 mm in MGBM vs. 1.8 mm in Pendulum; *p* = 0.01), without significant differences in distal tipping of molars. The MGBM group also showed greater vertical control of the first upper molars (Δ PP-16C: −0.4 mm in MGBM vs. 2.1 mm in Pendulum) and greater intrusion of the incisors (Δ PP-is: −0.8 mm MGBM vs. 1.4 mm Pendulum).

## Discussion

The purpose of this retrospective study was to compare the treatment effects of the MGBM system and Pendulum to look for significant statistical and clinical differences between the two systems in terms of dentoalveolar and skeletal effects. The Pendulum appliance was chosen for the control group because it is one of the most thoroughly investigated non-compliance distalizing appliances in the literature [3,4]. Maxillary molars in both groups were distalized successfully to class I relationships without patient cooperation. The MGBM protocol emerged, on the basis of average distalization time and the amount of molar distal movement recorded, as the more clinically effective and efficient of the two treatment modalities.

These findings support those recorded in the review done by Fudalej and Antoszewska [5] of studies on orthodontic distalizers reinforced with temporary skeletal anchorage. Indeed, the mean distal movement of the maxillary molars ranged from 3.5 to 6.4 mm. Antonorakis [12] reported a mean 3.1 mm of molar movement produced by conventional distalizing appliances featuring palatal force application and 2.6 mm for those with buccal force application.

In our study, we found that although, with the MGBM system, forces are applied on the buccal side, the skeletal anchorage allows a larger amount of posterior displacement compared to the conventional anchorage. Similar results were reported by Gelgor et al. [13] in 2007, who compared buccal and palatal distalizing mechanics reinforced with skeletal anchorage. The authors suggested that these results might be due to the presence of increased frictional forces on molar derotation in palatal distalizing mechanics.

Distalization appliances should ideally provide a bodily distal displacement of the molar. The amount of molar tipping has been found to vary greatly, depending on the device used: Antonarakis and Kiliaridis [12] reported distal tipping of 8.3° for devices with buccal application of force versus 3.6° for devices with palatal application of force.

On the other hand, Fuziy et al. [14], using the Pendulum, reported a molar distal movement of 4.6 mm and a distal tipping of 18°, while Mossaz et al. [15], using the same appliance, reported a distal movement of 3.8 mm and a distal tipping of 2.9°. In our study, the maxillary first molars were tipped distally by 9° (PP) in the MGBM group and by 9.8° in the Pendulum group. However, the differences in distal tipping between the two groups were not significant.

Many authors found tipping occurring as a result of distalization by distalizers reinforced with TADs. The review of Fudalej et al. [5] reported molar distal tipping ranging from 0.80° to 12.20°. In both the MGBM and the Pendulum groups, the distal and reaction force vectors were found to be located lower than the molar center of resistance, which means that the distal movement of a molar is accompanied by a distal tipping of the tooth.

The main disadvantage of distalizing procedures, common to all intraoral distalization appliances, is the forward movement of the anchoring teeth. The resulting loss of anchorage is expressed by mesial movement of the premolar and incisor segment. As reported by Feldmann and Bondemark [16], anchorage loss measured at the incisors or premolars ranged from 0.2 to 2.2 mm, and the anchorage loss/distal molar movement ratio ranged from 0.2 to 0.8 mm.

Surprisingly, in the present study, no significant differences were found between the two groups when measuring anchorage loss at the premolar level. The skeletal anchorage provided for by the MGBM protocol does not completely eliminate the loss of anterior anchorage. There are various possible reasons for this. As reported by Kinzinger et al. [17] and Liou et al. [18], palatal miniscrews may, due to absence of osseointegration and the elasticity of the bone, show small movements when stressed by orthodontic forces. Another important factor to consider is the reduced stiffness of the anchoring system to which the miniscrews are connected. The wire ligatures connecting the miniscrew to the transpalatal bar are elastic, as is the transpalatal bar. The unexpected loss of anterior anchorage could also be explained by the distance between the point of buccal application of force and the center of resistance of the tooth. In the review by Antonarakis and Kiliaridis [12], it was shown that devices with palatal application of distalizing forces showed less anchorage loss and therefore less anterior movement of the premolars (1.3 vs. 2 mm) compared to those using buccal mechanics. During molar distalization, the upper incisors are proclined and moved forward as a result of reaction forces that act first on the premolars before being transmitted to the incisor region. In our study, the maxillary incisors in the Pendulum group exhibited significantly more flaring during molar distalization. Indeed, the anterior movement of the anchorage unit was found to be reduced when the reaction forces of the distalization system are able to download directly onto a palatal miniscrew, as this reduces mesial force vectors on both the first and the second premolars. In fact, these teeth, when they are not anchored to distalizing system, follow in part the distal movement of molars under the influence of trans-septal fibers. In this way, the loss of anchorage in premolars can be eliminated, increasing spontaneous distalization.

At the end of the distalization, the MGBM group showed significantly less anchorage loss measured at the incisors, which resulted in significantly less proclination than found in the Pendulum group. Our findings are in agreement with those of Kinzinger et al. [17] who, utilizing a distal jet supported by two palatally inserted miniscrews, reported a mean anchorage loss of 0.71 ± 0.78 mm at the level of the first premolar and 0.36 ± 0.32 mm at the level of the central incisors.

In our study, significant differences in vertical movements of distalized molars were found between the two groups. The buccal distalizing force of the MGBM system resulted in a greater extrusive action on the first molars compared with that produced by the Pendulum appliance. Our findings seem to support those of Antonarakis et al. [12], who reported that distalizing devices with forces applied on the palatal side seem to cause less extrusion of molars, but more marked vertical changes at the level of the premolars and incisors compared to devices with buccal application of force.

Probably as a result of the greater molar extrusion and greater amount of distalization and molar rotation, we found a significantly greater decrease in the SNB angle for the MGBM compared with the Pendulum group. However, these effects proved transitory, as demonstrated by the cephalometric measurements recorded at the end of the fixed appliance therapy (T2). In fact, in the T1-T2 phase, there was greater compensatory counterclockwise rotation of the occlusal plane and the mandibular plane in the MGBM group compared to the Pendulum group, accompanied by a more pronounced forward projection of the pogonion. These findings could be explained by a greater amount of vertical growth in the condyle area to ‘compensate’ for the increasing vertical dimension of the dentoalveolar region. As a result, there was no clockwise rotation of the mandible, and point B, at the end of the treatment, was in a position favorable to a good esthetic outcome.

Analyzing the two samples throughout the observation period (T0-T2), we found that correction of molar relationship occurred in both groups with no significant differences in terms of sagittal and vertical skeletal changes. The only exception concerned the A point, which was found to be significantly more forward projected in the MGBM group.

## Conclusions

Based on the above discussion, the following conclusions were deduced:

- The MGBM system and the Pendulum appliance are both effective in the correction of class II malocclusions.
- The MGBM system was found to be more efficient than the Pendulum, producing greater distalization in a shorter treatment time.
- A certain amount of anchorage loss occurred in both groups even though the MGBM protocol made provision for skeletal anchorage.

## Abbreviations

Co: condilion
Po: porion
S: sella
Ba: basion
Pt: pterygoid point
Or: orbital
N: nasion
En: tip of nose
UL: superior labial point
LL: inferior labial point
Xi: mandibular centroid
ANS: anterior nasal spine
PNS: posterior nasal spine
A: subnasal point
B: sovramental point
Pm: paramedian point
Pg: pogonion
Pgc: soft pogonion
Gn: gnathion
Me: menton
Go: gonion
ii: lower incisor point
is: superior incisor point
iia: apical point of lower incisor
isa: apical point of superior incisors
ms: mesial point of upper first molar
mi: mesial point of lower first molar
NSL: sella-nasion line
PP: palatal plane
PM: mandibular plane
OL: occlusal plane
OLp: perpendicular occlusal plane passing for sella point
Ba-Na: cranio-basal plane
Pt-Gn: facial axis
E-plane: esthetic plane
N-Pg: facial plane
